# SARS-CoV-2: International Investigation Under the WHO or BWC

**DOI:** 10.3389/fpubh.2021.636679

**Published:** 2022-02-03

**Authors:** Mirko Himmel, Stefan Frey

**Affiliations:** ^1^Department for Microbiology and Biotechnology, Institute for Plant Sciences and Microbiology, University of Hamburg, Hamburg, Germany; ^2^Bundeswehr Research Institute for Protective Technologies and CBRN Protection, Munster, Germany

**Keywords:** SARS-CoV-2, COVID-19, BWC, forensic investigation, transparency measures, WHO, UNSGM, UN

## Abstract

In late 2019, the novel and highly infectious coronavirus SARS-CoV-2 caused a worldwide outbreak of a severe respiratory infectious disease, known as COVID-19. The disease has started in China and turned into one of the worst pandemics in human history. Due to the very fast global spread of the pathogen, COVID-19 is a great challenge for the Public Health Systems. It had led to a variety of severe limitations in private and public life worldwide. There is a lively public debate about possible sources of SARS-CoV-2. This article aims at providing a better understanding of controversial biological and political issues regarding COVID-19. Recommendations are made for possible actions under the umbrella of the World Health Organization and in respect to the Biological Weapons Convention.

## Introduction

In December 2019, an outbreak of an unknown viral pneumonia was reported in the city of Wuhan, capital of China's central Hubei province (1). The virus was identified as severe acute respiratory syndrome coronavirus 2 (SARS-CoV-2) which belongs to the virus family of *Coronaviridae* (2) and causes the disease commonly known as *Coronavirus Disease* 2019 (COVID-19). Since its official reporting in December 2019 globally, more than 273 Mio cases and more than 5.35 Mio COVID-19 related deaths (as of December 20, 2021) are registered (3). Therefore, COVID-19 has the potential to become one of the most severe and fatal pandemic disease to date (4). The World Health Organization (WHO) declared COVID-19 in February 2020 as a Public Health Emergency of International Concern. Additionally, due to its numerous negative effects on physical and mental health, social well-being, on economies and societies leading to the exacerbation of inequalities within and between countries the Seventy-third World Health Assembly expressed its concerns about the global pandemic (5).

In general, the COVID-19 pandemic is responsible for a variety of severe limitations and changes in private and public life worldwide. Shrinking economies were an immediate result from induced lockdowns, which led to a rise in unemployment and therefore decreasing demands of consumer goods. For example, the world trade had a steep decline in the first half of the year 2020 because the merchandise trade indicating a year-on-year drop of around 18.5%. According to the World Trade Organization this was caused by the coronavirus pandemic and the associated lockdown measures in many countries (6). Fortunately, after recovering in the second half of 2020 the decrease of the international merchandise trade volume was only 5.3% in 2020 (7). In the second semester of 2021 the world merchandise trade underwent a rebound, though varying among regions, exceeding the experts' predictions (8).

The drastic restrictions of public life, education and business activities in combination with a deliberately spread of misleading information about COVID-19. All this together led to concerned citizens and civil unrest in many countries. Some people might even dive into conspiracy theories believing the deliberate release of the virus as one possibility (9). Adding to that, unsubstituted claims were made that the virus might be engineered and part of a clandestine Chinese Bioweapons Program. At the center of these speculations is the Wuhan Institute for Virology (WIV), which is located close to the wet-market where COVID-19 might have its origin. Undoubtedly, the WIV is a well-known research institute with a strong focus in coronavirus research. However, there is reason to believe that the WIV could have been involved in secret Chinese military projects due to its relations to Chinese military researchers. Of note, there are no proofs available in the open literature that such a program does exist (10, 11). In part, these speculations were fueled by the fact that initial reports of an unusual pneumonia in Wuhan were obviously suppressed by local Chinese authorities (12–14). On September 12, 2019, the WIV database containing information on collected virus strains and genome sequences was removed from the internet/open-access (15).

Consequently, there were calls for an international investigation to identify the source of the COVID-19 outbreak (16, 17). However, it is not yet clear under which organizational umbrella such an investigation could take place and which institution could perform an internationally acceptable unbiased investigation regarding the causes of this pandemic. The intensive debate about acceptable conditions for a WHO-China joint investigation (finally performed early in 2021, see below) highlight these shortcomings of neutral investigation conditions even more.

In this policy brief, we discuss the role of the WHO as a first point of contact for an investigation of the source of the COVID-19 pandemic and possible roles of the Biological and Toxin Weapons Convention (BWC). Scientific publications, media reports, and official governmental statements were analyzed to investigate possible solutions for strengthening public health responses to the COVID-19 pandemic. A special focus was put on instruments discussed within the BWC regime and the academic biosecurity community. Possible investigation scenarios based on current international accepted mechanisms and initial steps how to investigate the source of a pandemic like COVID-19 were also elaborated.

### Critical Questions About the Origins of the SARS-CoV-2 Outbreak

Several publications conclude that SARS-CoV-2 originated from nature and was not man-made or released accidently from a research laboratory (18–22). Nevertheless, the SARS-CoV-2 outbreak raised questions if:

i. the outbreak was a consequence of a laboratory accident?
Scientific reports have documented a few accidental coronavirus releases from Chinese laboratories in the past (23–25). In 2004, a local outbreak of SARS-CoV-1 was reported in China due to laboratory-acquired infections (LAI). Chinese governmental authorities quickly contained these outbreaks (26). In a WHO report from May 2004, it states that “WHO and Chinese authorities view with concern the occurrence of laboratory-associated SARS cases.” (27). Publications that are more recent express their worries about inadequate biosafety management systems, insufficient resources for efficient laboratory operation, deficiency of professional capacity and a missing open culture in connection with this biosafety level (BSL) 4 laboratory (28, 29). LAI-infected personnel or staff performing environmental sampling of potentially infected bats, all being part of research activities conducted by the WIV, could cause the spread of the virus.
ii. SARS-CoV-2 is a genetically engineered virus?
One of the most urgent questions related to the COVID-19 pandemic was, if this infectious disease outbreak was caused by a genetically modified coronavirus. Scientists at WIV indeed conducted extensive research projects on coronaviruses including virus strains closely related to SARS-CoV-2. They used standard methods in virus cell culture as well as genetic engineering. Several manuscripts were published including so-called gain-of-function (GOF) experiments (30–33). Currently, the Congress of the United States is investigating the National Institute of Allergy and Infectious Diseases (NIAID) grant project number R01AI110964. This grant was awarded to Eco Health a US non-profit organization which funded Coronavirus-research at the WIV as a sub-grantee (34). Within this research project, additional GOF experiments were carried out (32). Both, the NIAID and the Eco Health risk assessments of the proposed GOF experiments did not seem to reflect the required balance between risks and benefit (35). Detailed investigations of SARS-CoV-2 genomes have revealed two notable regions of interest within the spike protein-coding gene:

1) The ACE2 receptor-binding domain of the spike protein: This protein is exposed to the viroid surface and acts as a ligand for the host cell angiotensin-converting enzyme 2 (ACE2) receptor. Binding of the viral spike protein to the ACE2 receptor is a key step for virus entry into the host cell (36, 37).
2) The second notable region is a furin cleavage site, which is located at the junction of S1 and S2 subunit of the spike unit. This peptide insertion is involved in the proteolytic cleavage of the spike protein. Enveloped viruses like the coronaviruses require proteolytic cleavage of the spike unit to be able to infect the host cell (38, 39). SARS-CoV-2 has a unique polybasic cleavage site (RRAR) which could influence transmissibility and host range (40). Only a few other coronaviruses are known for having different amino acid sequence motifs of the proteolytic cleavage site (41, 42).

iii. SARS-CoV-2 was already circulating for longer, but infections were not made public.
There are also reports pointing to a much earlier occurrence of SARS-CoV-2 than December 2019, as has been communicated by the Chinese health authorities. There was one case reported from a 55-year-old resident from Hubei province published in the South China Morning Post on 17 November 2019 (43, 44). Further laboratory-confirmed COVID-19 cases in humans which did not have had direct contact to the Wuhan city wet market, were reported starting from December 1, 2019 (45). In France, the first patient known to be infected with the pandemic coronavirus (“index patient”) was hospitalized in December 2019 (46). Therefore, the virus might have circulated for much longer. In October 2019, some athletes who attended the Military World Games went sick with symptoms similar to COVID-19 (47, 48). Chinese authorities dated the earliest known COVID-19 case back to the beginning of November 2019.
Interestingly, a possible precursor virus related to SARS-CoV-2 could be the causative agent for the infection of six mineworkers in Mojiang, Yunnan province, in April 2012 (49, 50). These workers got severely ill showing symptoms attributed to a SARS-CoV-1 infection. Subsequently a coronavirus infection was later confirmed by the WIV (51). From 2012 to 2015, WIV scientist has collected annual samples of bats in the same cave in which the six mineworkers got infected (51, 52) This theory proposes the possibility that the origin of the SARS-CoV-2 virus could be from in the WIV stored samples taken from theses infected Mojiang Miners.

In sum, there is scientific evidence pointing to a natural spillover of SARS-CoV-2, but there is also other evidence supporting manmade sources of the COVID-19 pandemic. Therefore, it is important to investigate thoroughly the COVID-19 outbreak on a sound scientific basis [see for example the critics of Graner et al. (53) of the publication from Holmes et al. (22)]. In the following section, we first analyze possible ways for an international investigation of the COVID-19 outbreak under the umbrella of the WHO, before we examine potential contributions of procedures linked to the BWC regime.

## Policy Options and Implications

### Investigation Under the Responsibility of WHO, the First Step

**Figure 1:**
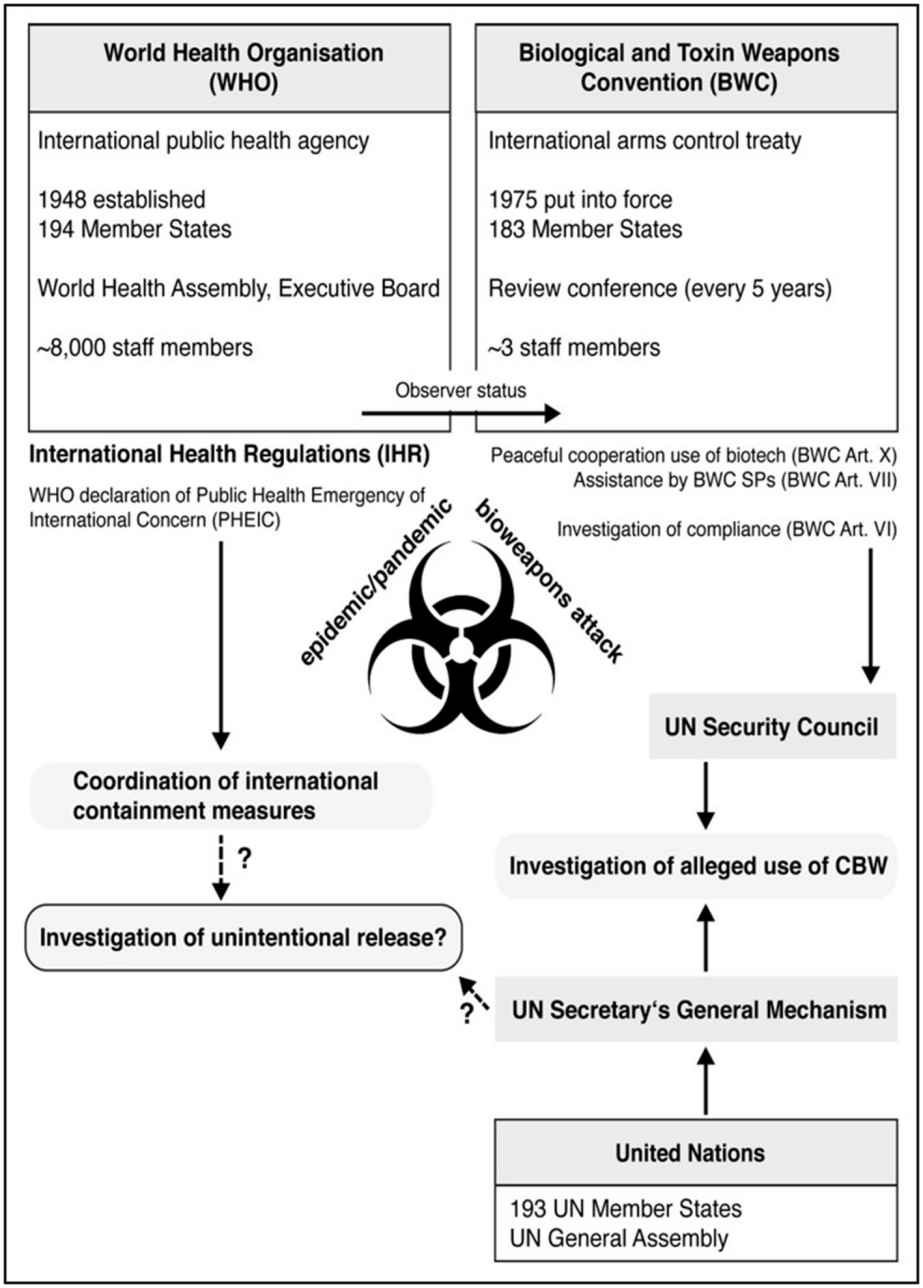
Possible interplay between WHO and BWC investigative mechanisms. Shown are key features of both, the WHO and the BWC regime including status of membership and decision-making bodies. Although acting in different areas (WHO, human health; BWC, biological arms control), there might be an interplay between internationally agreed investigative mechanisms usable for the analysis of the COVID-19 outbreak.

Which international organization could be taken responsible for the unambiguous investigation of a pandemic outbreak? Independently obtained results of an investigation would ideally be recognized by the international community as a whole. The United Nations (UN) as the largest and most powerful international organization represents 193 countries in the world. In general, the UN could initiate such an investigation (Figure 1) (54). As a specialized agency of the UN, the WHO's primary function is to promote human health globally. Several times in the past, the WHO has played a leading role in eradication of infectious diseases especially by supporting vaccination campaigns.

End of January and mid of February 2020, two WHO-China Joint Missions were carried out. During the first mission, visits of different facilities in Hubei province were conducted. Furthermore, ongoing epidemiological surveillance processes were analyzed. Infection prevention and control measures as well as the deployment of a RT-qPCR test kit for the detection of the new coronavirus were discussed with Chinese officials (55). Epidemiological aspects, response and preparedness measures, containment and collaborative programs were investigated during the second mission (56). The goal of the second mission was to inform about the national and international planning of the WHO regarding the following steps to improve readiness and preparedness for non-affected COVID-19 areas. Nevertheless, there was no bioforensic investigation of the COVID-19 outbreak carried out by an independent research team. In May 2020, the WHO Emergency Committee recommended a joint investigation to be conducted by experts for human and animal health. This joint investigation should also aim the rapid identification of the zoonotic source of SARS-CoV-2 (57). This could trigger a third WHO-China Joint Mission making use of modern bioforensic methods. As there are currently no international guidelines for forensic investigations of a pandemic, lessons learned from this field mission could be used by the WHO experts to prepare a draft guideline for future investigations. The International Health Regulations (IHR) are designed to prevent or control the spread of diseases and they providing guidance for adequate public health response (58). At the seventy-third World Health Assembly (WHA), first steps were decided to improve countermeasures required for the containment of COVID-19 (published in resolution WHA73.1, pages 6 and 7 (5). In July 2020, the inauguration of an independent Panel for Pandemic Preparedness and Response (IPPR) was announced by the WHO Director-General, which will evaluate the response to the COVID-19 pandemic, globally (59). Further efforts have been made in August 2020 to examine the effectiveness of the IHR and to prepare the ground for necessary amendments (60). In reaction to increasing public pressure, the Chinese government agreed to host a Joint WHO-China Study from 14 January to 10 February 2021. Aim of this mission was the analysis of potential zoonotic sources of SARS-CoV-2 and the search for intermediate hosts of this virus. The multidisciplinary team of Chinese and international experts performed several investigations on the ground in Wuhan and presented key findings in a final report (61) The report received critics due to the lack of firm data supporting the conclusions presented, for example ruling out a lab accident as “extremely unlikely” (62). In a press statement, the WHO Director-General later made clear that investigations of the origins of SARS-CoV-2 must go on (63).

The COVID-19 pandemic could act as a model for efficient international assistance and future cooperation in the public health sector and beyond. What emerged evident during the first months of this pandemic is the need for improvements in communication and better-synchronized containment measures by all countries worldwide. In this respect, the delayed reporting of human-to-human spreading of the disease did cost valuable time (several weeks) before appropriate health protection measures such as social distancing, masks, and isolation of clusters of infected people were put into action. It should be noted that the time required for efficient disease containment measures mandatory for an efficient infectious disease containment might be quite reasonable. This holds even truer in cases where asymptomatic disease carriers already shed sufficient amounts of the virus to initiate new chains of infection. Here, an improved epidemiological – and less political – driven reporting system would allow to keep pace with such dynamic disease outbreaks. In the following section, we contemplate investigative procedures linked to the BWC regime.

### Investigation of the COVID-19 Pandemic in Respect to Procedures of the BWC

The Biological and Toxins Weapons Convention (BWC) of 1972 is a multilateral arms control treaty banning the development, production, stockpiling and - in addition to the Geneva Protocol of 1925 - use of biological weapons (BW) (Figure 1). Currently, 183 UN countries are Member States of the BWC. Although there are no indications for a deliberate release of SARS-CoV-2, the BWC regime offers a framework for a coordinated reaction by BWC Member States on the question of the origin of the COVID-19 pandemic.

In particular, Article VII of the Convention^1^ calls upon States Parties for mutual assistance in the case of a biological weapons (BW) attack. Required resources and capacities for detection and diagnosis of natural occurring pathogens and toxins, which could be provided by at least some BWC Member States, are also well suitable for analyzing unexpected disease outbreaks. Furthermore, measures for counteracting against the use of BW could also be applicable for containing an epidemic and could help to prevent a possible international public health crisis. Suitable measures include large-scale quarantine, transport and hospitalization of severe cases across national borders or the deployment of medical emergency teams in affected countries. Mass vaccination programs, the *ad hoc* establishment of large-scale medical infrastructure like field hospitals, and the installation of field laboratories could be used to improve public health systems responses to severe epidemics/pandemics. But most importantly, BWC Member States offering assistance to a country challenged by a disastrous biological event or incident is meant to enhance confidence among States Parties.

In 2020, United Nations Secretary-General António Guterres highlighted the danger of a deliberate use of diseases as weapons at a Security Council video conference. Especially, diseases that were deliberately altered to be more virulent or intentional released are of concern, as we are not yet prepared to deal with such viruses on a global scale. The Secretary-General also commented on the requirement of a verification mechanism to implement confidence-building measures within the framework of the convention. The BWC regime should be better prepared to cope with upcoming biological threats by improved preventative measures, enhanced response capacities and effective countermeasures (64).

In case of an alleged use of biological warfare agents, the UN Security Council (UNSC) should have the obligation to take action and to initiate an investigation. However, in the current political situation the UNSC often has difficulties to find consensus on urgent questions related to human safety and security. For example, there is no common understanding within the UNSC on procedures to investigate alleged chemical weapons attacks, e.g., during the Syrian civil war.

Due to the absence of any legally binding verification mechanism within the BWC regime there are no procedures implemented *en detail* on how BWC States Parties should act in the event of an abusive spread of pathogens. In the case of alleged use of biological weapons States Parties could approach the UN Security Council (UNSC) and asks for investigation of the complaints, but this procedure has never been evoked and given the political tensions frequently seen in the UNSC it appears difficult to predict the outcome of such an appeal. Negotiations of BWC verification protocol failed in 2001 and currently there are no clear indications for a restart of this process. It would be more than desirable to see an impulse set by the COVID-19 pandemic for BWC Member States to again engage in strengthening of the BWC. At the 2021 BWC Meeting of Experts, the US Under Secretary of State for Arms Control and International Security provided ideas for such a move forward in her statement^2^. The Ninth Review Conference of the BWC in August 2022 offers the opportunity for Member States to actively address the above-mentioned challenges in a cooperative manner.

The amendment of the BWC by an additional protocol would be another possibility to provide States Parties to react fast and efficient on incidents related to possible uses of BW. Such an addendum could regulate standardized investigative measures and, if necessary, sanctions or obligations of the international community in such an event.

Another approach is the UN Secretary-General's Mechanism (UNSGM) mechanism, which was established as a verification instrument of the Geneva Protocol for investigating an alleged use of chemical and biological weapons. UN General Assembly established this mechanism with resolution A/RES/42/37 C (1987). The UN Secretary-General can react on appeals also by single UN Member States in the case of alleged use of chemical and biological weapons. Several Member States of the BWC consider the UNSGM also as a valuable tool for an investigation of unusual outbreaks of infectious diseases in case of a potential alleged use as a bioweapon. Although the UNSGM is currently the key instrument in the international toolbox of the investigation of unusual biological incidents, it is limited to the investigation of the alleged use of bioweapons. In comparison to the strict quality standards within the investigations of chemical weapons, no quality standards are existing for biological weapons. In case of an investigation of the alleged use of a potential bioweapon laboratory results might be therefore easily questioned or even rejected.

The idea of activating the UNSGM is not accepted by all BWC States Parties, therefore its final authority to investigate biological incidents would be highly controversial. In 2020 and 2021, for example, Member States of the BWC have attempted to weaken the UNSGM and the power of the UN Secretary-General by submitting working papers and voting on their contents in the First Committee of the UN General Assembly. If a biological event was caused deliberately must therefore be assessed with high confidence before the UNSGM is applied to prevent political damage to this investigative instrument. However, the public debate about the origin of SARS-CoV-2 shows how difficult this might be to achieve. In order to further investigate the possibility of an incidential release of SARS-CoV-2 a transparent investigation would be required. Research at the WIV was partly funded in by the US money and conducted in collaboration with US scientists.

In the light of this complex situation, free international scientific exchange of scientists is essential, which is also supported by Article X of the BWC calling upon States Parties to cooperate for the peaceful use of biotechnology. In order to strengthen and cultivate scientific friendships in the areas of relevance to the BWC, a framework should be established. Within this framework scientists could meet and interact more closely – inspired by political questions related to the BWC, but not dominated by political tensions.

Cooperation of States under the WHO umbrella to further investigate the origins of SARS-CoV-2 appears to be the most intriguing way forward. Elements of the BWC regime might be give orientation on how to further proceed. In the following, we provide recommendations for an internationally agreeable investigation of the COVID-19 outbreak.

## Actionable Recommendations

### Investigation Under the Responsibility of WHO

Desirably, in agreement with all WHO Member States, a multidisciplinary international task force could be established within a new mandate to take medical, environmental, and biomedical samples in states parties of interest, e.g. China. This task force would be required to obtain and study available information relating to these allegations including interviews. It could further perform or delegate (with the support of the UNSGM laboratory network) the analysis of the beforehand acquired samples. Unfortunately, during the WHO-China joint investigation in 2021, limited capacity to investigate thoroughly resulted in unattainable objectives. In the future, the demand to agree on an mutual accepted mandate will also include a specific framework in which to operate successfully. Moreover, it must be clarified to what extend a country, by the time of an outbreak, would have the obligation to fully support such an investigation. The implementation of an internationally agreed procedure for a WHO investigative mechanism is for sure a most difficult endeavor. Strengthening public health and swift support as well as transparent investigation of severe infectious disease outbreaks should be investigated while considering the responsibility of all states regarding a sustainable global health. However, it would also be important that investigations into a pandemic origin would include a joint effort between WHO member states and experts from the country of interest. These measures could ensure a basis of trust. In addition, the support of the investigation team might assist affected countries with disease surveillance measures. The discussion of an investigated outcome will be held on a neutral and professional level and it must always be assumed that the presumption of innocence applies. If need for action is identified e.g. the improvement of essential laboratory infrastructure and the implementation of an appropriate biosafety and biosecurity management, it should be promoted within the international community (65).

### Approaches for Forensic Investigations Under the BWC

In the BWC regime, there is still an ongoing political debate about the requirement for a verification mechanism. Nevertheless, BWC Member States should take every necessary step to further extend the agreed confidence-building measures by novel approaches including technical means. Another relevant aspect could be fostering international scientific cooperation under the umbrella of the Convention. In this respect, the COVID-19 pandemic could be a trigger for such activities. Clearly, a naturally occurring infectious disease outbreak is not a matter for the BWC. But treaty members frequently confirmed that capabilities for the detection of biological weapons attacks must be in place before such an incident might take place^3^. Adequate methods of microbial forensics useable to investigate the alleged use of biological weapons could also be applied for the analysis of the source of the COVID-19 pandemic (66, 67). This could be done either by agreement of BWC Member States to use these approaches in a combined effort, by the activation of the UNSGM triggered upon request by UN Member States or by decision of the UNSC to mandate such investigation. The latter two might be politically difficult to achieve depending on the political circumstance. However, the first option is based on experiences taken from the reaction of a couple of BWC Member States during the Ebola virus outbreak 2014–2016 in West Africa (68).

In the hypothetical case of the unintended release of a pathogen from a laboratory or biotechnological installation subsequently leading to an epidemic/pandemic, it is of utmost importance to quickly identify the corresponding facility. A network of governmental laboratories working at BSL-3 or even BSL-4 level would be of help. A database of research capabilities of these labs could be established. Database entries should focus on biosafety and biosecurity assessments of the work but would give no exact details about sensitive information in order to respect intellectual property and national security concerns. Network members could provide guidance how to monitor research activities of the listed labs, for example, in the context of a still-to-come Scientific Advisory Board of the BWC. Moreover, such mechanism could improve international standards in biosafety and biosecurity and ultimately support an open scientific exchange within the international community (69). Building trust among BWC Member States is an essential step, especially in the light of ongoing debate, about the (im-) possibility of the verification of the BWC. This is even more true, since some treaty Member States fear violations of intellectual property as well as negative impacts on national security by an intrusive verification mechanism. Industrial laboratories could also be included at a later stage, as confidence building has progressed. In addition, this might even be an appropriate way to implement a verification system within the BWC.

This process could be started by organizing scientific conferences organized by a UN organization (e.g., UNODA), which would be focused on various fora for a rather informal personal exchange between scientific and technical experts, politicians and diplomats. The idea is that there are no diplomatic constraints within the discussions of scientists. The annual BWC Meeting of Experts could be used as template for such conference, which would be held readily in advance to the next BWC Review Conference. At the scientific conference and in support by a newly founded science advisory board of the BWC working groups could be set up. Tasks of relevance to the BWC would include assessing the impact of new (bio-)technologies, requirements for internationally agreed safety and security standards in biological laboratories. Furthermore, internationally agreed standard procedures for the epidemiological and bioforensic investigation of outbreaks caused by the potential misuse of pathogens.

The mechanisms outlined above would allow for an improved open exchange on developments in science and technology between the three BWC regional groups (Western Group, East European Group and Non-Aligned Movement). Precondition for the proposed measures is, of course, the political will to further develop the BWC regime in a cooperative manner. Nevertheless, an enhanced scientific exchange along with better opportunities for less well-equipped BWC States Parties to participate and benefit from this process might increase the political commitment to the BWC regime including the required financial support.

## Conclusions

The COVID-19 pandemic exposes frictions in the international system to counteract biological threats. Loss of confidence in internationally agreed mechanisms for reporting, monitoring and management of epidemics/pandemics is of great concern, especially for the WHO. Lack in transparency and delayed reporting of key epidemiological and biological data by some states in the course of the emerging COVID-19 pandemic clearly showed the need for improving international mechanisms counteracting biological threats. In the view of the current tense political situation, which is marked by mutual allegations of inappropriate COVID-19 countermeasures and negligent inadequate monitoring measures in biosafety and biosecurity between leading countries, the return to cooperative action for an unbiased SARS-CoV-2 outbreak analysis following scientific standards is highly desirable. In this respect, re-strengthening of the role of the WHO and its investigative mechanisms would be of utmost importance. The BWC, being primarily an arms control treaty, offers rather an outline than detailed practical steps to be taken for the analysis of unusual infectious disease outbreaks. Instruments such as the UNSGM could provide valuable tools for performing required scientific and technical analyses without necessarily triggering the mechanism. Nevertheless, there is still no internationally accepted running workflow how to perform the bioforensic investigation of pandemic outbreaks. The international community should take the responsibility for improving and protection global public health by activating relevant political instruments, which are designed for that very purpose.

## Author Contributions

All authors listed have made a substantial, direct, and intellectual contribution to the work and approved it for publication.

## Conflict of Interest

The authors declare that the research was conducted in the absence of any commercial or financial relationships that could be construed as a potential conflict of interest.

## Publisher's Note

All claims expressed in this article are solely those of the authors and do not necessarily represent those of their affiliated organizations, or those of the publisher, the editors and the reviewers. Any product that may be evaluated in this article, or claim that may be made by its manufacturer, is not guaranteed or endorsed by the publisher.

## Footnotes

^1^BWC Article VII: “Each State Party to this Convention undertakes to provide or support assistance, in accordance with the United Nations Charter, to any Party to the Convention which so requests, if the Security Council decides that such Party has been exposed to danger as a result of violation of the Convention”.

^2^Ambassador Bonnie D. Jenkins stated that “[The Ninth BWC Review Conference] should establish a new expert working group to examine possible measures to strengthen implementation of the Convention, increase transparency, and enhance assurance of compliance. […] It could be useful to build on some approaches suggested in past discussions, but our efforts should not be defined by them.” Available at: https://geneva.usmission.gov/2021/11/22/remarks-to-the-2021-biological-weapons-convention-meeting-of-states-parties/ (Accessed: November 27, 2021).

^3^“States Parties noted the importance of ensuring that efforts undertaken are effective irrespective of whether a disease outbreak is naturally occurring or deliberately caused [...]. States Parties also recognized that capabilities to detect, quickly and effectively respond to, and recover from the alleged use of a biological or toxin weapon need to be in place before they are required.” Report of the BWC Meeting of States Parties in 2010.
